# Two-step single-reactor synthesis of oleic acid- or undecylenic acid-stabilized magnetic nanoparticles by thermal decomposition

**DOI:** 10.3762/bjnano.14.2

**Published:** 2023-01-03

**Authors:** Mykhailo Nahorniak, Pamela Pasetto, Jean-Marc Greneche, Volodymyr Samaryk, Sandy Auguste, Anthony Rousseau, Nataliya Nosova, Serhii Varvarenko

**Affiliations:** 1 Organic Chemistry department, Lviv Polytechnic National University, Bandera street 12, 79013, Lviv, Ukraine; 2 Institute of Macromolecular Chemistry, Czech Academy of Sciences, Heyrovského nám. 2, 162 06 Prague 6, Czech Republic; 3 Institut des Molécules et Matériaux du Mans (IMMM), UMR 6283 CNRS − Le Mans Université, Avenue Olivier Messiaen, 72085 Le Mans Cedex, France

**Keywords:** Fe(III) acetylacetonate, iron oxide nanoparticles, maghemite, magnetic nanoparticles, magnetite, thermal decomposition synthesis

## Abstract

Different iron oxides (i.e., magnetite, maghemite, goethite, wüstite), particularly nanosized particles, show distinct effects on living organisms. Thus, it is of primary importance for their biomedical applications that the morphology and phase-structural state of these materials are investigated. The aim of this work was to obtain magnetic nanoparticles in a single reactor using Fe(III) acetylacetonate as the initial precursor for the synthesis of Fe(III) oleate or Fe(III) undecylate followed by their thermolysis in situ. We proposed a new approach, according to which the essential magnetite precursor (a complex salt of higher acids – Fe(III) alkanoates) is obtained in a solvent with a high boiling point via displacement reaction of acetylacetone with a higher acid from Fe(III) acetylacetonate during its elimination from the reaction mixture under vacuum conditions. Magnetic nanoparticles (NPM) were characterized in terms of morphology, hydrodynamic diameter, and composition via several techniques, such as transmission electron microscopy, dynamic light scattering, thermogravimetric analysis, Fourier-transform infrared spectroscopy/attenuated total reflectance, ^57^Fe Mössbauer spectroscopy, and X-ray diffraction. The effect of unsaturated oleic (OA) and undecylenic (UA) acids, which are both used as a reagent and as a nanoparticle stabilizer, as well as the influence of their ratio to Fe(III) acetylacetonate on the properties of particles were investigated. Stable dispersions of NPM were obtained in 1-octadecene within the OA or UA ratio from 3.3 mol to 1 mol of acetylacetonate and up to 5.5 mol/mol. Below the mentioned limit, NPM dispersions were colloidally unstable, and at higher ratios no NPM were formed which could be precipitated by an applied magnetic field. Monodisperse nanoparticles of iron oxides were synthesized with a diameter of 8–13 nm and 11–16 nm using OA and UA, respectively. The organic shell that enables the particle to be dispersed in organic media, in the case of oleic acid, covers their inorganic core only with a layer similar to the monomolecular layer, whereas the undecylenic acid forms a thicker layer, which is 65% of the particle mass. The result is a significantly different resistance to oxidation of the nanoparticle inorganic cores. The core of the particles synthesized using oleic acid is composed of more than 90% of maghemite. When undecylenic acid is used for the synthesis, the core is composed of 75% of magnetite.

## Introduction

Magnetic nanoparticles are increasingly being used in various fields thanks to the recent progress in their controlled synthesis and knowledge of their chemical and physical properties. One such area is biomedicine [1]. Especially iron oxide-based nanoparticles, due to their biodegradation, low toxicity, and enhanced oxidative resistance compared to metallic nanoparticles, show high potential in biomedical applications [2–4]. Up to now, iron oxide nanoparticles have been proposed as contrast agents for magnetic resonance imaging, high-precision biosensors, and carriers in magnetic-assisted drug delivery systems. Furthermore, they are used for tumor treatment via the hyperthermia method and in bone tissue regenerative medicine [5–6].

However, using iron oxide nanoparticles in biomedicine requires in-depth studies of their structure and properties. It was well-established that different iron oxides (e.g., magnetite (Fe_3_O_4_), maghemite (γ-Fe_2_O_3_), goethite (α-FeOOH), and wüstite (FeO)), have a divergent impact on biological objects [7]. In this regard, studying the morphology and phase composition of iron-oxide-based nanoparticles is a critical issue. Nevertheless, magnetite and maghemite particles remain the most commonly used nanoparticles in biomedical applications. However, it must be noted that magnetite nanoparticles undergo rapid oxidation in air, leading to maghemite layer formation on their surface. The oxidation is significantly enhanced in the case of nanoparticles characterized by a large specific surface area. Thus it can be concluded that the smaller the nanoparticles, the more important the problem of protecting the surface of the magnetite particles from oxidation. Moreover, the magnetic properties of magnetic nanoparticles (NPM) significantly depend on their size [8]. Iron oxide nanoparticles (>100 nm in size) are typically multidomain and ferromagnetic, whereas nanoparticles (<100 nm in size) are usually single domain [9]. The further reduction of nanoparticle diameter below the critical size of 25 nm leads to nanoparticles with superparamagnetic properties [10–11]. Due to the absence of coercive forces in superparamagnetic nanoparticles not exposed to an external magnetic field, they are characterized by good colloidal stability, which makes them ideal candidates for magnetic-assisted targeted drug delivery [12].

Nanoscale magnetite can be obtained through well-known synthesis routes, such as hydrothermal synthesis, thermal decomposition, or co-precipitation [10–11]. Each of these synthetic approaches has certain advantages and disadvantages. One of the essential issues in many biomedical applications is the synthesis of magnetic nanoparticles with uniform size, chemical composition, and superparamagnetic properties. These requirements can be met by applying thermal decomposition, which is based on the decay of organic iron salts with low stability (i.e., acetate, pentacarbonyl, acetylacetonate) in high-boiling-point solvents in the presence of stabilizing agents (e.g., fatty acids, higher amines, alcohols) or their mixtures [13–15]. Although thermal decomposition is a relatively simple method, minor modifications of the reaction conditions (e.g., time, temperature, purity, or ratio of reagents) considerably influence the properties of the obtained nanoparticles [16]. Besides the narrow size distribution of the particles obtained by thermal decomposition, particle preparation with various morphologies (e.g., spherical, cubic, octahedral) is another great advantage of this method [17–18].

It has been previously reported that by using oleylamine as a stabilizer, the decomposition of thermally unstable Fe(III) acetylacetonate was observed at temperatures above 150 °C, followed by the formation of 10–16 nm FeO/Fe_3_O_4_ nanoparticles [19–20]. To synthesize magnetite nanoparticles, an additional component (e.g., 1,2-hexadecandiol) was introduced into the system [21]. According to the most common method, a dispersion of magnetite nanoparticles is obtained via thermolysis of commercial or separately synthesized Fe(III) oleate at 250–320 °C in the presence of oleic acid and a solvent with a high boiling point [22]. We found no publications about the two-step single-reactor synthesis of iron oxide NPM by thermal decomposition in which Fe(III) acetylacetonate can be used as starting compound for the synthesis of alkanoates followed by their thermolysis in solution.

This work aims to develop the synthesis of NPM dispersions by thermolysis of Fe(III) oleate or Fe(III) undecylate in a high-boiling-point solvent. Iron oxide nanoparticle dispersions were obtained via two-stage single-reactor synthesis with Fe(III) acetylacetonate as the initial precursor. The properties of the synthesized materials were studied using various methods.

## Results and Discussion

The physicochemical properties of NPM synthesized via thermal decomposition depend on many factors, such as selection of precursors and organic stabilizers, ligand/precursor ratio, solvent, and temperature of the decomposition reaction. Table 1 shows the results of synthesized nanoparticles obtained by variation of solvents and stabilizing agents. All prepared dispersions of the nanoparticles were initially black but slowly turned reddish upon exposure to air [15]. When the synthesis was carried out at a slight excess of fatty acids (oleic acid, OA, or undecylenic acid, UA), the unstable dispersions of particles were obtained, which coagulated and formed a black magnetic precipitate on the reactor walls (sample MT-III; initial precursor/OA molar ratio of 1:3.05). By applying the Fe(III) acetylacetonate/OA molar ratio of 1:3.29, partially stable dispersions of nanoparticles (samples MT-I and MT-VI) were obtained. When a significant excess of UA stabilizing agent was used (sample TMU-III, molar ratio above 1:5.5), a brown liquid without particles or magnetic sediments was observed.

**Table 1:** Synthesis conditions and characteristics of the obtained NPM.

Sample name	Stabilizer	Fe(III) acetylacetonate to carboxylic acid ratio (mol/mol)	Solvent	*T* (°C)	Stable dispersion	Size, *D* (nm)			*I*_PCR_^b^	Polydispersity index, PDI

						TEM	DLS	XRD^a^		

MT-I	OA	1:3.29	diphenyl	255	±	7.5–12.5	^c^	8.8	0.85–1.4	–
			
MT-II		1:3.37	paraffin		+	12^d^	^c^	8.8^c^	1.4	^c^
MT-III		1:3.05			–	–	–	–	–	–
MT-IV		1:3.30^e^			+	13	26	5.5	2.4	0.149
MT-VI		1:3.29			±	17^c^	^c^	8.8^c^	1.9^c^	–
		
TMO-I		1:3.30	1-octadecene	310	+	8	14	4.6 (6.0^f^)	1.7 (1.3^f^)	0.089
TMO-III		1:5.60			–	–	–	–	–	–
	
TMU-II	UA	1:3.50			+	13	34	6.9	1.9	0.242
TMU-III		1:5.80			–	–	–	–	–	–
TMU-IV		1:5.11			+	11	27	4.5	2.4	0.138
TMU-V		1:3.32			+	16	42	5.6	2.9	0.121

^a^According to the Scherrer's formula [23]; ^b^the polycrystallinity index calculated by the formula *I*_PCR_^= ^*D*_TEM_/*D*_XRD_ [24]; ^c^a significant number of aggregates does not allow the analysis; ^d^irregular nonspherical particles; ^e^two-fold lower concentration of the reagents; ^f^with Rietveld refinement.

According to the developed method, the formation of NPM has several stages. At the first stage of the synthesis, the change of ligands into Fe(III) acetylacetonate occurs. In the presence of higher carboxylic acids, such as oleic acid or undecylenic acid, Fe(III) alkanoate is a predominant product from Fe(III) acetylacetonate decomposition at a relatively low temperature range of 110–120 °C (see Figure S1 in Supporting Information File 1) [25]. The reaction of the ligand replacement is an equilibrium reaction. Therefore, acetylacetone must be removed from the reaction zone to shift the equilibrium towards the formation of the complex with OA or UA. To avoid the influence of acetylacetone residues formed during the decomposition of acetylacetonate, they were removed from the reaction mixture under vacuum. In this way, an early ineffective decomposition of iron acetylacetonate and the formation of oleate or undecylate can be avoided. There is a big difference between the boiling temperatures of acetylacetone (140.5 °С) and the higher acids used in the reaction (360 and 275 °C of OA and UA, respectively), and their losses during vacuuming are minimal.

In the case of incomplete removal of acetylacetonate ligands, the reaction mixture that undergoes thermolysis contains mixed ligands. The thermal decomposition of such salts at the second stage can significantly affect the processes of particle nucleation, composition, and morphology [26–27].

To estimate the completeness of oleate and formation of undecylate complexes, Fourier-transform infrared spectroscopy (FTIR) studies of the reaction mixtures were carried out at the beginning and at the end of the first stage of NPM synthesis prior to removing acetylacetone under vacuum (see Figures S2 and S3 in Supporting Information File 1).

The spectra of iron(III) acetylacetonate, 1-octadecene, oleic or undecylenic acid, and the reaction mixture at different synthesis stages were compared (see S0 Stage1, SFin Stage1 in Figures S2 and S3, Supporting Information File 1). All spectra of iron(ІІІ) acetylacetonate, 1-octadecene, oleic acid, and undecylenic acid pure substances agree with those described in the literature [28–29].

At the beginning of the first stage (S0 Stage1 for each of the acids), one can observe peaks of iron(III) acetylacetonate in the spectra. The vibration bands of the carboxylate group ν_a_ (COO^–^) are visible at 1577 and 1524 cm^−1^ and a band at 432 cm^−1^ corresponds to the vibrations of Fe–O or С–СН_3_ [29–30].

At the end of the first stage (SFin Stage1), the absorption bands corresponding to the acetylacetonate ligand or acetylacetone residues are not observed. The absorption band at 432 cm^−1^ also disappears, which indicates that it has been correctly attributed to C–CH_3_ fragments [30].

The IR bands characteristic of metal carboxylates are in the range of 1650–1500 cm^−1^ for the asymmetrical vibrations and 1450–1300 cm^−1^ for the symmetrical vibrations. The difference between the bands ν(COO^–^) (∆ = CO_asym_ − CO_sym_) in the region 1300–1650 cm^−1^ can be used to determine the carboxylate coordination mode. For ∆ > 200 cm^−1^, a monodentate ligand is expected, whereas for ∆ < 110 cm^−1^, a bidentate ligand is expected. For a bridging ligand, ∆ is between 110 and 200 cm^−1^ [31].

Spectra of the complexes (see SFin Stage1, Figure S2 and Figure S3 in Supporting Information File 1) formed due to the exchange reaction of acetylacetonate and a higher acid show several absorption bands which were absent in the spectra of reactants and 1-octadecene (Table 2).

**Table 2:** Absorption bands of oleate and undecylate Fe(III) complexes formed after decomposition of iron(III) acetylacetonate in the presence of a given amount of a higher acid (OA or UA) in 1-octadecene.

Entry	Iron(III) oleate complexes, cm^−1^	Iron(III) undecylate complexes, cm^−1^	assignment

1	1741	1742	C=O str, monomer
2	1711	1712	C=O str, dimer form (acycl. or cycl.)
3	1599	1600	ν_a_(COO^–^)
4	1538	1552	ν_a_(COO^–^)
5	1443^a^	1443^a^	ν_s_(COO^–^)
6	1416^a^	1417^a^	ν_s_(COO^–^)

^a^Assumed according to the literature [22,25].

The low-intensity absorption band at 1741–1742 cm^−1^, which appeared in the spectra of the reaction mixture of both oleate and undecylate complexes in octadecene, corresponds to vibrations of the carbonyl group of free unassociated acid. This band is not observed in the spectra of pure acids. The absorption band at 1711 cm^−1^ in both cases can be attributed to stretching vibrations of C=O in one of the types of (cyclic or acyclic) dimer binding observed for the majority of aliphatic carboxylic acids in nonpolar environments [32]. It is also confirmed the absence of the absorption band of the OH group at 3300–3500 cm^−1^ and the presence of the absorption band at 2677–2688 cm^−1^, referring to –OH stretching oleic/undecylenic acids in dimer form in the spectra of OA and UA (see Figure S2, Figure S3, respectively, in Supporting Information File 1) [33]. In the reaction mixture spectra at the end of the first stage of the synthesis, the intensity of the band at 1711 cm^−1^ is reduced compared to that at the beginning of this stage (spectra S0 Stage1) due to the consumption of acid and formation of a complex with Fe(III). There are two bands in the zone of asymmetric vibrations at 1500–1600 cm^−1^: for oleic complexes at 1599 and 1538 cm^−1^ and for undecylenic complexes at 1600 and 1552 cm^−1^.

The zone of symmetric vibrations of the carboxylate group ν_s_(COO^–^) of both oleate and undecylate complexes cannot be precisely determined because of the overlapping of different type absorption bands in 1-octadecene. However, according to the literature, it can be observed in the range of 1406–1440 cm^−1^ (most often at 1436 cm^−1^ and 1415 cm^−1^) [4,7]. In any case, the calculated difference for the oleic complex can be ∆ = 1599 − 1436 = 163 cm^−1^ and ∆ = 1538 − 1436 = 102 cm^−1^ or ∆ = 1599 − 1415 = 184 cm^−1^ and ∆ = 1538 − 1415 = 123 cm^−1^. Thus, values in the range of 200 > ∆ > 110 allow us to assume that the substitution of ligands in iron acetylacetonate of the formed oleate complex leads to the bridging type of coordination with some features of the bidentate type. Using this assumption for the undecylate complex, the difference is ∆ = 1600 − 1436 = 164 cm^−1^ and ∆ = 1552 − 1436 = 116 cm^−1^ or ∆ = 1600 − 1415 = 185 cm^−1^ and ∆ = 1552 − 1415 = 137 cm^−1^. The calculated values confirm the bridging coordination in this complex.

According to IR data at the beginning of the second stage of NPM synthesis (thermolysis at higher temperatures), the reaction mixture consists of 1-octadecene with dissolved ferric oleate or undecylate and the given amount of oleic or undecylenic acid (according to the synthesis conditions). These acids act as principal stabilizing agents in the formation of nanoparticles.

**Figure 1:**
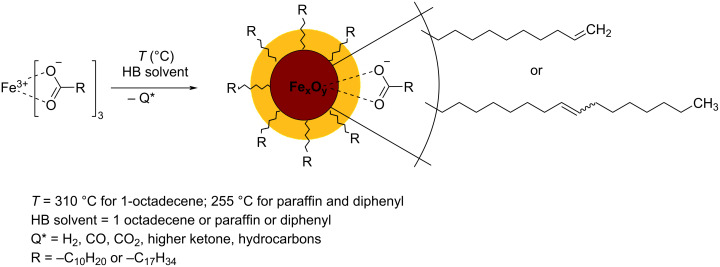
Formation of nanoparticles via decarboxylation of Fe(III) alkenoates.

Thermal decomposition of Fe(III) alkanoate at temperatures below 200 °C occurs at a negligible rate; thus, its impact is insignificant [34]. At temperatures above 200 °C, (310 °C in the case of 1-octadecene and 255 °C for diphenyl and paraffin), Fe(III) alkanoate undergoes decarboxylation thermolysis accompanied by breaking of the FeO–C bonds. The release of carbon mono- and dioxide, hydrogen, higher ketones, and hydrocarbons, as well as partial reduction of Fe(III) to Fe(II), results in the formation of magnetic iron oxide nanoparticles [35]. Excess of higher carboxylic acid that has not been bound to the iron salt, did not undergo thermolysis, and is likely to be adsorbed on the surface of the particles, ensures nanodispersion stability (Figure 1) [31].

**Figure 2:**
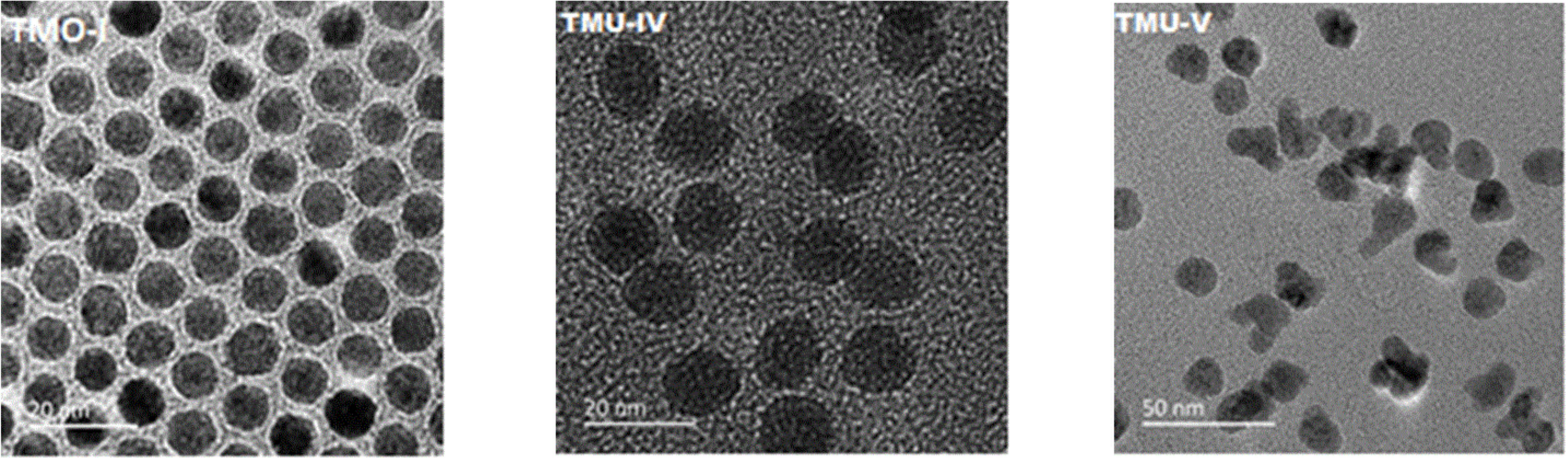
TEM micrographs of the nanoparticles synthesized in 1-octadecene using different stabilizers (TMO-I – OA, TMU-IV, TMU-V – UA).

**Figure 3:**
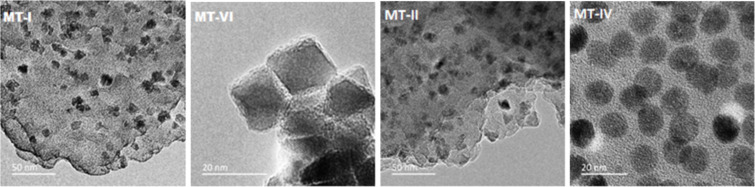
TEM micrographs of the nanoparticles synthesized using OA in paraffin (МТ-ІІ, MT-IV, MT-VI) and diphenyl (МТ-І).

Transmission electron microscopy (TEM) micrographs of the prepared nanoparticles confirmed their size between 8 and 16 nm (Figure 2 and Figure 3).

**Figure 4:**
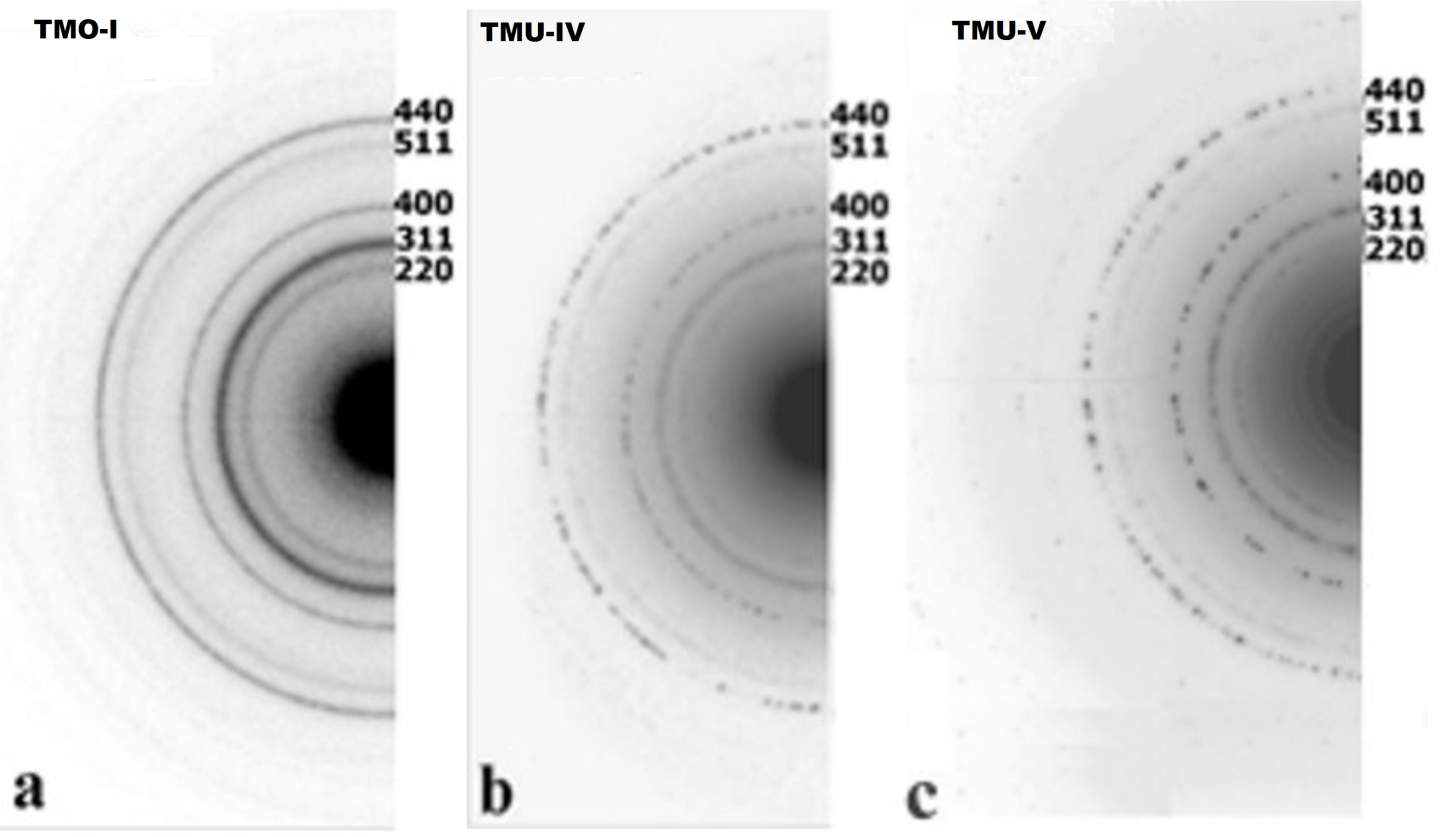
Electron diffraction patterns of (a) TMO-І, (b) TMU-IV, and (c) TMU-V samples.

The diameter and shape of the nanoparticles depend on the preparation conditions, especially the selection of higher (18 and 11 carbon atoms) fatty acids – the OA-stabilized nanoparticles were significantly smaller (8–13 nm) compared to UA-stabilized ones (11–16 nm). Furthermore, highly monodispersed spherical nanoparticles creating stable toluene dispersions, were obtained only when 1-octadecene was used as a solvent (samples TMO-I and TMU-IV, Figure 2 and Table 1). Most of the nanoparticles synthesized in paraffin and diphenyl were poorly reproducible and nonuniform in size and shape. Only the MT-IV nanoparticles, synthesized at a concentration twice as low as that of the reagents in paraffin compared to the rest of the synthetic approaches, were spherical (Figure 3). However, the yield of this synthesis, which was estimated in terms of maghemite according to the results of thermogravimetric analysis (TGA), was significantly lower compared to those conducted in 1-octadecene. The electron diffraction patterns (Figure 4) of the crystallographic planes present the Miller indexes as selected area electron diffraction (SAED) patterns and clearly correspond to a spinel phase, typical for both magnetite and maghemite.

Moreover, the fact that the halos observed were uniform and the single spots were not visible proved that the crystallites were very small. These results correspond well with data from X-ray diffraction (XRD), according to which the average size of the crystallites for all prepared nanoparticles was 4.5–9 nm. The average crystallite size did not correlate with the amount of stabilizer used. However, a large excess of NPM stabilizer was not obtained (samples TMO-III, TMU-III). The Rietveld refinement allowed us to determine a coherent diffracting domain size nanoparticle of 6 nm by using LaB_6_ as a reference compound (Table 1). The particularly good agreement of indices (*R*_exp_ = 0.44, *R*_p_ = 1.17, *R*_wp_ = 1.75, *R*_Bragg_ = 24.23, and GoF = 15.55) confirmed a proper refinement. The size of 8–13 nm was determined from the TEM microphotographs of the particles synthesized using OA. This value is actually closer to the average size of the crystallites obtained by estimating the expansion of the X-ray diffraction line (DXRD calculated with Scherer, optionally Rietveld, refinement), which indicated a single magnetic domain characteristic of the TMO-I nanoparticle sample. When a stabilizer with a shorter carbon chain (i.e., UA) is used under the same synthesis conditions, particles of larger sizes (11–16 nm) are formed. These particles are characterized by a significantly higher polycrystalline index (*I*_PCR_ = 1.9–2.9) and it seems that they are aggregates of smaller crystallites. This result also correlates with a higher polydispersity index observed for these nanoparticles (Table 1).

**Figure 5:**
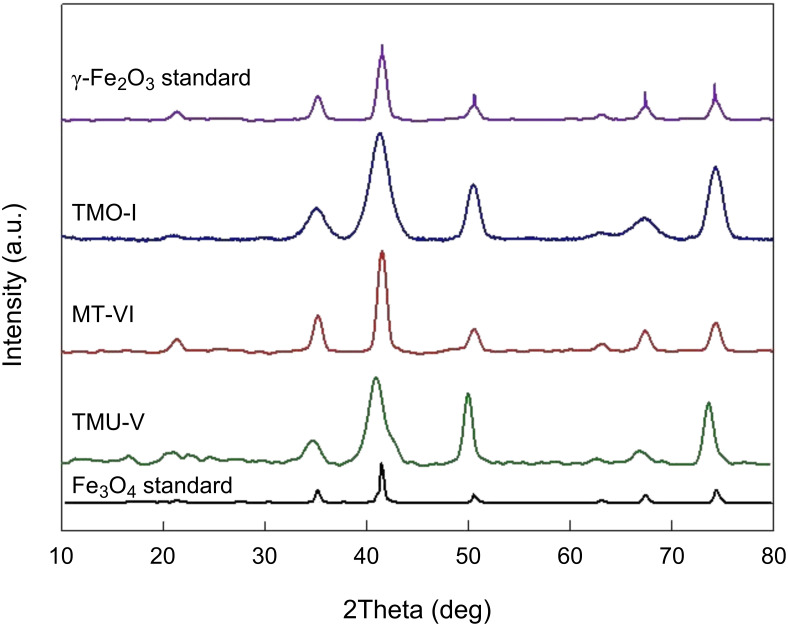
X-ray diffraction patterns of (1) Fe_3_O_4_ standard (JCPDS No. 88-315; mean crystallites size of 11 nm) [1], (2) TMU-V, (3) MT-VI, (4) TMO-І, and (5) γ-Fe_2_O_3_ standard (JCPDS No.00-039-1346) [36–37].

The X-ray diffraction patterns of the synthesized nanoparticles (magnetite and maghemite) were compared to those of standard nanoparticles (Figure 5).

The diffraction patterns of the synthesized nanoparticles showed significant similarity to the patterns of the standards. However, it must be noted that these iron oxides are characterized by spinal structures and very close lattice parameters, which makes their distinction using XRD very troublesome [38].

**Figure 6:**
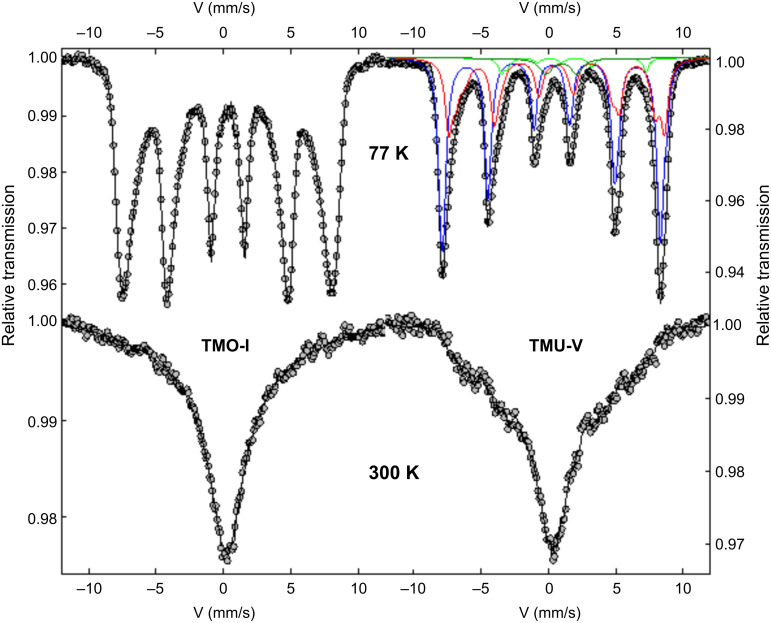
Mössbauer spectra of the samples TMO-I and TMU-V at 300 and 77 K (blue, red, green, and olive green correspond to Fe^3+^, intermediate Fe^3+^–Fe^2+^, Fe^2+^ magnetic components, and quadrupolar Fe^2+^ component, respectively).

Unlike XRD, ^57^Fe Mössbauer spectroscopy allows one to distinguish between magnetite and maghemite, since the isomeric shift resulting from the monopolar electric interaction is very sensitive to the valence states of Fe. Taking into account the characteristic measurement time of ^57^Fe Mössbauer spectroscopy, estimated at 10^−8^ s at the Larmor frequency, the ultrafine structure of magnetite at 300 K (and above the Verwey transition, estimated at 119 K) consists of one-third of Fe^3+^ parts and two-thirds of Fe^2.5+^ species according to its expected electronic and stoichiometric structure. The Mössbauer spectra of the TMO-І nanoparticle sample at 300 and 77 K are shown in Figure 6. At 300 K, a broadened single line typical for the presence of superparamagnetic relaxation phenomena suggested a very small size (about 10 nm compared to results from the literature) for the synthesized nanoparticles, which was consistent with electron and XRD diffraction results, as well as TEM results.

Different fitting models can be considered using either a distribution of hyperfine fields or a superposition of two separate lines, resulting in an invariant mean of the isomeric shift, which corresponds to the presence of only Fe^3+^ moieties. In contrast, the Mössbauer spectrum recorded at 77 K consisting of a pure symmetric magnetic sextet is composed of broadened and asymmetric lines. It can be well described by a discrete distribution of hyperfine fields, which also leads to an average isomeric shift corresponding to the presence of only Fe^3+^ moieties. Thus, it can be concluded that the described nanoparticles are monodisperse and composed of maghemite only. According to previous studies, magnetite–maghemite core–shell nanoparticles can be prepared by partial oxidation of magnetite core during and/or after synthesis, and the thickness of its shell can be controlled. Furthermore, the prolonged oxidation may result in the production of maghemite nanoparticles. These observations agree with the result of the present study, confirming the monodisperse nature of the maghemite nanoparticles obtained.

The TMU-V nanoparticle sample was also measured at 300 and 77 K (Figure 6), and the profiles differ from those observed for the TMO-I sample. The shape of a single line at 300 K and a more pronounced ultrafine structure at 77 K indicate a decrease in superparamagnetic relaxation phenomena, probably caused by a larger size of the nanoparticles. Furthermore, the spectrum recorded at 77 K can be divided into four different components: (i) three magnetic components attributed to blocked Fe^3+^ moieties, blocked Fe^2+^ moieties, and Fe ions with intermediate valence state between Fe^3+^ and Fe^2+^, as commonly observed in typical magnetite below the Verwey transition; (ii) a quadrupolar component assigned to Fe^2+^ moieties, probably due to some traces (4%) of FeO (wüstite). However, the values of ultrafine parameters, in particular average values of isomer shifts (regardless of the fitting model), indicate a mixed-phase composition of the nanoparticles (i.e., maghemite:magnetite in a ratio of 25:75) according to the Fe atomic proportions, thereby proving their partial oxidation [39–40].

**Figure 7:**
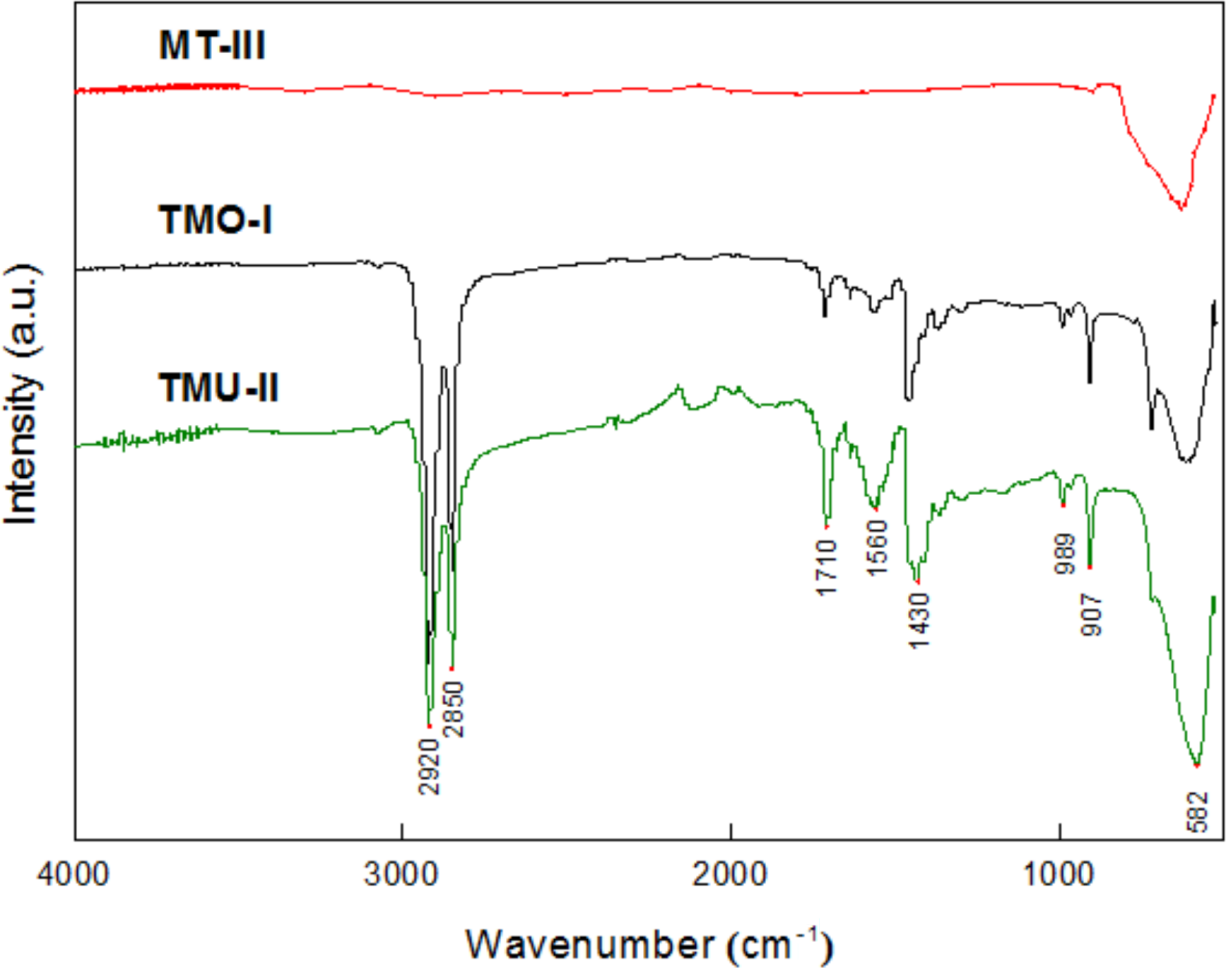
ATR-FTIR spectra of NPM samples stabilized with 1) OA (TMO-I), 2) UA (TMU-ІІ), and 3) iron oxide powder without stabilizers (MT-III).

The Fourier-transform infrared spectroscopy/attenuated total reflectance (ATR-FTIR) and TGA techniques were used for surface characterization in terms of the presence of higher fatty acids, which play an essential role in the stability and future functionality of the particles. ATR-FTIR studies confirmed the presence of characteristic bands that could be assigned to the organic (stabilizing) layer on the surface of the nanoparticles synthesized with both OA (TMO-I) and UA (TMU-II) (Figure 7).

The peaks at 2850 and 2920 cm^−1^ were attributed to symmetric and asymmetric stretching vibrations of CH_2_ groups of carboxylic acid. The band at 1710 cm^−1^ corresponds to vibrations of the C=O group in one of the types of (cyclic or acyclic) dimeric binding, which is characteristic of most aliphatic carboxylic acids in non-polar environments [32]. The peaks at 1640, 1456, and 1377 cm^−1^ are ascribed to the deformation vibrations of isolated C=C bonds in OA and UA, whereas the bands at 1560 and 1430 cm^−1^ correspond to asymmetric and symmetric vibrations of COO^–^ groups of the stabilizing layer attached to the core of the nanoparticles. The difference of 130 cm^−1^ indicates that after thermolysis, the bridging type of coordination is maintained on the surface of the particles, the same as for the precursors (see Supporting Information File 1) [28]. The intense peak at 582 cm^−1^ is typical of the Fe–O vibrations.

**Figure 8:**
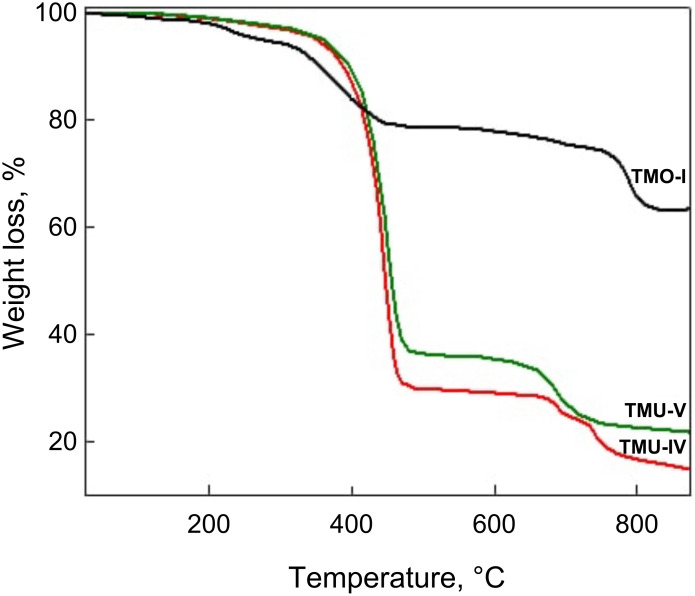
Thermogravimetric analysis of (1) TMO-I, (2) TMU-V, and (3) TMU-IV.

The thickness of the organic shell was estimated by comparing the hydrodynamic diameter of the particle and its size previously determined using TEM microphotographs (Table 1). For the UA-stabilized nanoparticles, the thickness of the organic layer was 8–13 nm, while for the OA-coated nanoparticles, it was 3–6 nm (TMO-I). These outcomes agreed with the TGA results, in which the nanoparticles synthesized under the same conditions but with divergent carboxylic acids contained significantly different amounts of the organic phase. Determined from the TGA curves, the content of the stabilizer for the samples TMO-I (coated with OA) and TMU-V (coated with UA) was 20.6% and 64.4%, respectively (Figure 8).

Moreover, taking into account the particle average diameter, densities of the particle core and organic shell, as well as the content of the organic phase, the average thickness of the stabilizer layer (H) was calculated (Equation 1), and the values of 3.1 nm for the sample TMO-I and 9.1 or 12.0 nm for the samples TMU-IV or TMU-V, respectively, were obtained. Based on the fact that the length of the OA molecule is 1.9–2.1 nm, it can be assumed that the core of the TMO-I particle is a coat with a monolayer of OA. In contrast, the shell thickness of the TMU-V particles significantly exceeded the length of the UA molecule (≈1.7 nm), indicating the formation of a multilayer shell.

## Conclusion

The developed method of Fe(III) acetylacetonate high-temperature decomposition in the presence of a higher unsaturated carboxylic acid (OA or UA), without additional co-stabilizers or reducing agents, could be applied to obtain monodisperse nanoparticles of iron oxide (Fe_3_O_4_, Fe_2_O_3_, or their mixtures) with controlled dimensions using the single-reactor synthesis, which involves the vacuum-controlled formation of Fe(III) alkanoates.

It was established that the most effective factor affecting the stability of dispersions and the properties of particles is the ratio of the high unsaturated acid to Fe(III) acetylacetonate. Stable dispersions of NPM in an aliphatic solvent were obtained in 1-octadecene within the ratio of OA or UA of 3.3–5.5 mol to 1 mol of acetylacetonate. The size of the particles and their resistance to oxidation depend on the type of higher unsaturated acid used. By using oleic acid, particles with a size of 8–13 nm are obtained, which are not capable of resisting oxidation processes and therefore consist of maghemite. In contrast to oleic acid, when using undecylenic acid in the synthesis, dispersions of magnetite particles of larger sizes (11–16 nm) are obtained, which have a large organic shell (up to 65% of the particle mass) protecting them from oxidation.

Such stable dispersions of functionalized nanoparticles can be used in biomedicine and in the production of composites with specified properties.

## Experimental

### Materials and methods

Fe(III) acetylacetonate (Fe(III) 2,4-pentadienoate, 97%), undecylenic acid (UA, 96%), paraffin (used after recrystallization), diphenyl (99%), 1-octadecene (91%), and propanone were purchased from Merck KGaA (Darmstadt, Germany). Oleic acid (OA, 98%) was bought from Lachema (Brno, Czech Republic). For magnetic separation, a permanent cylindrical neodymium magnet (NdFeB; 45 × 15 mm), with an induction on the surface of 1.2 T, was used. To characterize the size, morphology, hydrodynamic diameter, X-ray diffraction, transmission electron microscopy, and dynamic light scattering (Malvern Zetasizer Nano S, Palaiseau, France) were used. Transmission electron microscopy observations were conducted using a JEOL JEM 2100 HR microscope (Croissy Sur Seine, France) equipped with a LaB_6_ source, and an accelerating voltage of 200 kV was applied. The electronic diffraction patterns were obtained using the SAED technique. The XRD diffraction patterns were collected using a PANalytical MPD-PRO diffractometer equipped with a linear X’celerator detector and a Co Kα lamp as a source of radiation (1.789 Å). The experimental data were analyzed using the HighScorePlus software, with the implemented Rietveld method [41]. This method gives different types of crystallographic information, such as the size of the unit cell, the coordinates of the atoms, and the agreement indices which show a good refinement. The collected XRD patterns were compared with the standard of maghemite and magnetite available in the International Centre for Diffraction Database (ICDD).

Specific structural and magnetic properties of the synthesized NPM were studied using ^57^Fe Mössbauer spectroscopy at 77 K. The samples were investigated using a conventional transmission device with a Co source diffused into a Rh matrix. The hyperfine parameters were refined by using quadrupolar doublets and magnetic sextets with Lorentzian lines. The values of the isomer shift are quoted to that of α-Fe at room temperature (RT). Indeed, this technique is highly sensitive to the valence state of Fe species and thus enables the identification of magnetite and maghemite and the estimation of their respective proportions. Measurements were performed in a transmission geometry with a 925 MBq γ-source of ^57^Co/Rh mounted using a conventional constant acceleration. The velocity of the source was calibrated using α-Fe as the standard at RT. The measurement was performed on solid pellets made of dried nanoparticles containing approx. 5 mg Fe/cm^2^. The Mössbauer spectra were fitted using the MOSFIT program (the Modular Open Source Fitter for Transients, a Python 2.7/3.x package for fitting, sharing, and estimating the parameters of transients via user-contributed transient models) involving quadrupolar and magnetic components with Lorentzian lines. The isomer shift values are referred to as that of α-Fe at RT.

The ATR-FTIR measurements were performed on a ThermoScientific iD5 ATR Nicolet iS5 IR spectrometer (Waltham, MA USA) on a diamond crystal and Spectrum Two FTIR Spectrometer with Universal ATR (PerkinElmer, USA). The thermogravimetric measurements were carried out using a TA Instrument, Hi-Res-Dynamic TGA Q 500 (New Castle, USA) in a nitrogen atmosphere and in a temperature range of 25–900 °С (heating rate of 10 °C/min, N_2_ flux 80 mL/min). Before the TGA measurements, the nanoparticles were dried under vacuum at 50 °C.

The average particle size was processed using the ImageJ software.

The thickness of the organic shell of the stabilizer on the particle surface was calculated using TEM (*D*_TEM_) and TGA data according to Equation 1:


[1] H (nm)=ms(DTEM2)3⋅ρnmn⋅3⋅ρs3,

where *H* (nm) is the thickness of the layer of the organic shell on the surface of the inorganic core of the spherical particle; *m*_s_ is the ratio between the organic shell of the stabilizer on the surface and the mass of the particles (determined by the TGA method); *m*_n_ is the ratio of the inorganic core from the mass of the particles (determined by the TGA method); *D*_TEM_ is the particle diameter (determined by TEM); ρ_n_ is the core material density (magnetite ρ = 5.1 g/cm^3^, maghemite ρ = 4.9 g/cm^3^); and ρ_s_ is the density of the shell material (ρ_OA_ = 0.89 g/cm^3^ – OA, ρ_UA_ = 0.912 g/cm^3^ – UA).

### Synthesis of iron oxide nanoparticles

The synthesis of nanoparticles was carried out in a 100 mL three-necked glass reactor equipped with a reflux condenser and a mechanical stirrer. A Wood’s metal alloy bath with temperature control within 100–400 °C was used for heating. Fe(ІII) acetylacetonate (3 g, 8.49 mmol) was dissolved into the solvent of choice (30 mL; diphenyl, 1-octadecene, or paraffin). Different molar ratios of carboxylic acid (OA or UA) to Fe(III) acetylacetonate (in the ratio from 1:3.05 to 1:5.8) were used. The solution was heated to 120 °C and the acetylacetone (AcAc) was removed under a vacuum of 150 mmHg with constant stirring. Then, the reflux condenser was replaced by an air condenser, and the reaction mixture was heated to 255 °C in the case of diphenyl and paraffin and to 312 °C under argon, in the case of 1-octadecene. The stirring was continued for 30 min. The resulting reaction mass was transferred into a 250 mL reactor equipped with a mechanical stirrer and washed five times with propanone (150 mL each time) followed by nanoparticle deposition by magnetic separation on a NdFeB magnet under argon. The supernatant was eliminated by decantation, and the precipitate was suspended in 5 mL of hexane using an ultrasonic bath UM-2, 140 W (Olsztyn, Poland). The remains of propanone were removed under vacuum, and the resulting nanoparticles were resuspended in toluene. Then a stable colloidal solution of NPM was stored under argon.

## Supporting Information

File 1 Additional figures.
