# Supplementary material for: Two-step single-reactor synthesis of oleic acid- or undecylenic acid-stabilized magnetic nanoparticles by thermal decomposition
Source: Beilstein J Nanotechnol. 2023 Jan 3;14:11–22. doi: 10.3762/bjnano.14.2 (PMC9830496; doi:10.3762/bjnano.14.2)
Supplement: File 1 — Additional figures. [file Beilstein_J_Nanotechnol-14-11-s001.pdf]

BEILSTEIN JOURNAL OF NANOTECHNOLOGY

Supporting Information

for

Two-step single-reactor synthesis of oleic acid- or undecylenic acid-stabilized magnetic nanoparticles by thermal decomposition

Mykhailo Nahorniak, Pamela Pasetto, Jean-Marc Greneche, Volodymyr Samaryk, Sandy Auguste, Anthony Rousseau, Nataliya Nosova and Serhii Varvarenko

Beilstein J. Nanotechnol. **2023,** *14,* 11–22. doi:10.3762/bjnano.14.2

Additional figures

License and Terms: This is a supporting information file under the terms of the Creative Commons Attribution License (https://creativecommons.org/licenses/by/4.0). Please note that the reuse, redistribution and reproduction in particular requires that the author(s) and source are credited and that individual graphics may be subject to special legal provisions.
The license is subject to the *Beilstein Journal of Nanotechnology* terms and conditions: (https://www.beilstein-journals.org/bjnano/terms)

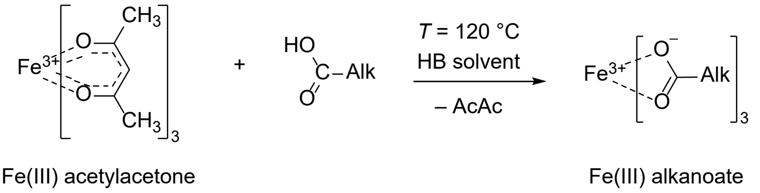

Figure S1: Fe(III) alkanoate is formed by the ligand exchange reaction of Fe(III) acetylacetonate in the presence of higher carboxylic acid. HB Solvent: 1-octadecene, paraffin or diphenyl.

Attenuated total reflection Fourier-transform infrared spectroscopy.

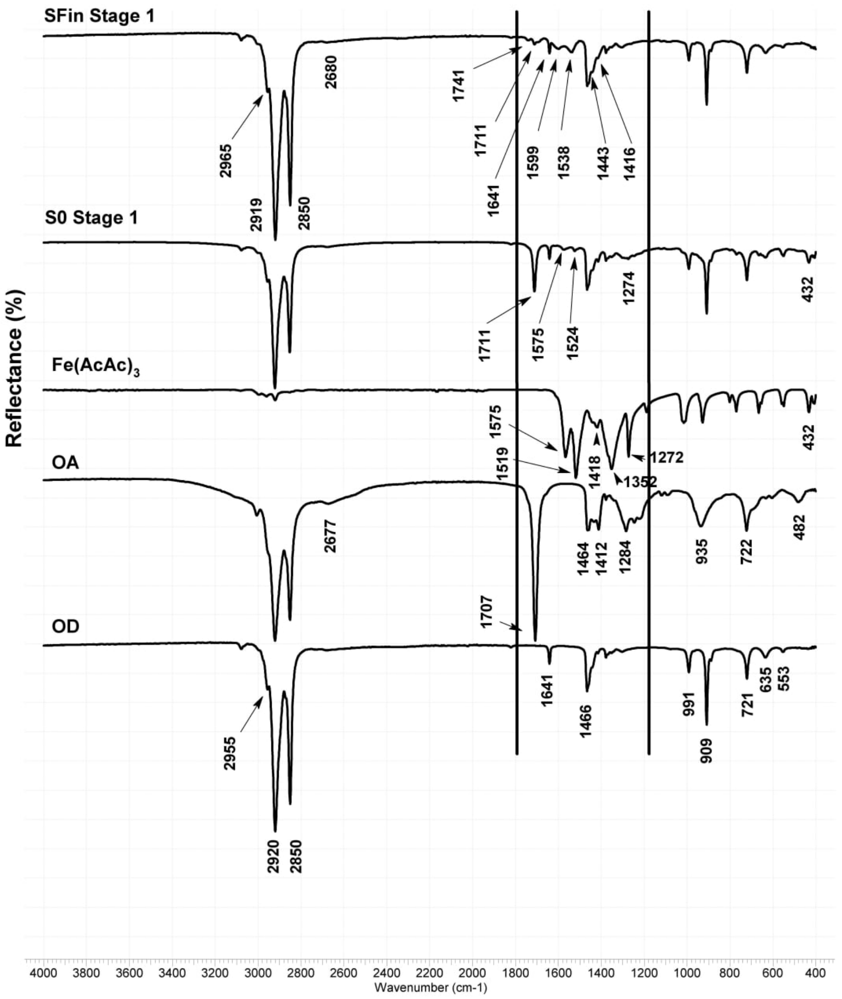

Figure S2: FTIR spectra: 1-octadecene (OD); oleic acid (OA); and Fe(III) acetylacetonate (Fe(AcAc)$_3$). Reaction mixture containing Fe(III) acetylacetonate and oleic acid in 1-octadecene at the beginning of the first stage of the synthesis of NPM prior to the removal of acetylacetone under vacuum (S0 Stage1); the reaction mixture containing Fe(III) oleate in octadecene at the end of the first stage of NPM synthesis after removal of acetylacetone under vacuum (SFin Stage1). The studies were conducted under the conditions of TMO-I sample synthesis.

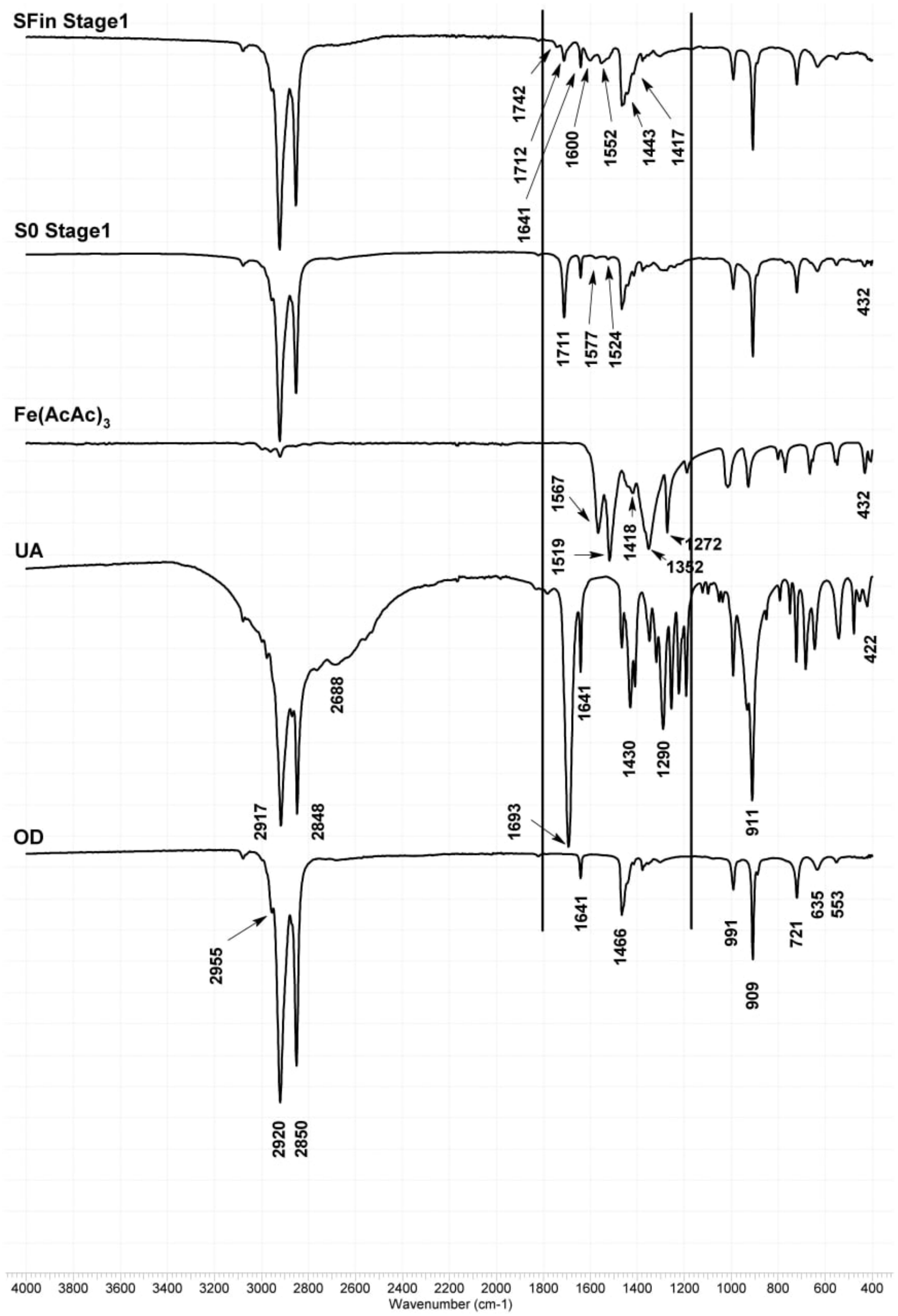

Figure S3: FTIR spectra: 1-octadecene (OD); undecylenic acid (UA); Fe(III) acetylacetonate (Fe(AcAc)$_3$). Reaction mixture containing Fe(III) acetylacetonate and undecylenic acid in 1-octadecene at the beginning of the first stage of the synthesis of NPM prior to the removal of acetylacetone under vacuum (S0 Stage1); the reaction mixture containing Fe(III) undecylate in octadecene at the end of the first stage of NPM synthesis after removal of acetylacetone under vacuum (SFin Stage1). The studies were conducted under the conditions of TMU-V sample synthesis.
